# The nasal symbiont *Staphylococcus species* restricts the transcription of SARS-CoV-2 entry factors in human nasal epithelium

**DOI:** 10.1016/j.isci.2021.103172

**Published:** 2021-09-25

**Authors:** Jeong-Yeon Ji, Ara Jo, Jina Won, Chan Hee Gil, Haeun Shin, Sujin Kim, Yung Jin Jeon, Hyun Jik Kim

**Affiliations:** 1 Department of Otorhinolaryngology, Seoul National University College of Medicine, 103 Daehak-ro, Jongno-gu, 110-799 Seoul, Korea; 2 Seoul National University Hospital, Seoul, Korea; 3 Department of Otorhinolaryngology, Gyeongsang National University Hospital, Jinju, Korea

**Keywords:** Biological sciences, Molecular biology, Microbiology, Virology

## Abstract

Emerging evidence indicates that severe acute respiratory syndrome-related coronavirus-2 (SARS-CoV-2) is transmitted through the human nasal mucosa via the principal entry factors angiotensin-converting enzyme 2 (ACE2) and transmembrane serine protease 2 (TMPRSS2), which are highly expressed in the nasal epithelium. Therefore, the biologics targeting host entry factors on human nasal mucosa will be necessary for complete control of SARS-CoV-2. Our data reveal that *ACE2* was more abundant in human nasal mucosa than lung tissue. Both *ACE2* and *TMPRSS2* transcriptions significantly decreased in nasal epithelium in response to *S. epidermidis* and were relatively lower in human nasal mucus with large numbers of *S. epidermidis*. *ACE2* transcription was also reduced in nasal epithelium in response to nasal symbiont *S. aureus*. This study proposes that *Staphylococcus species* nasal commensals might potentially restrict SARS-CoV-2 entry to the nasal epithelium via down regulation of cellular receptors coupled with reduction of principal host protease.

## Highlights

• The cellular receptor of SARS-CoV-2 was more distributed in human nasal mucosa
• Human nasal epithelium is the primary target of SARS-CoV-2 transmission
• *Staphylococcus species* reduce the transcription of SARS-CoV-2 entry factors
• Nasal symbionts impede the cellular invasion of SARS-CoV-2 into human nasal epithelium

## Graphical abstract

**Figure:**
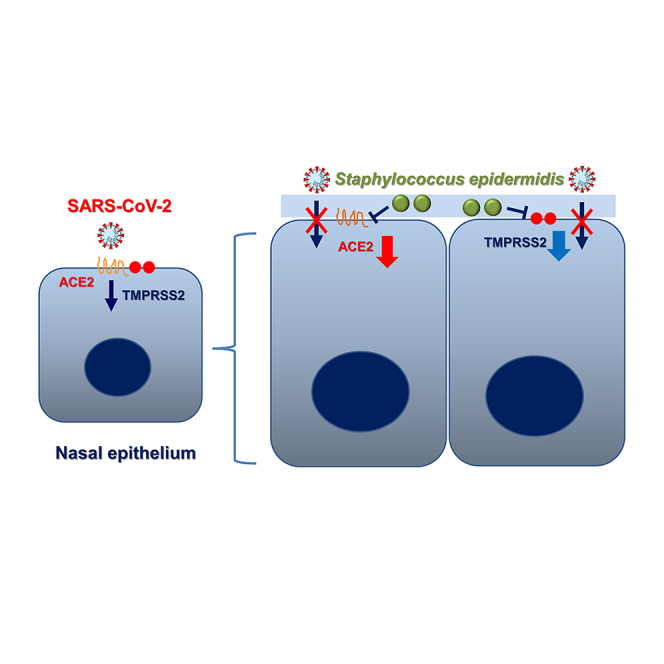

## Introduction

At present, the world is suffering from a pandemic infection of severe acute respiratory syndrome-related coronavirus-2 (SARS-CoV-2), which causes coronavirus disease 2019 (COVID-19) and leads to acute respiratory distress syndrome or viral pneumonia with severe damage to the lungs (Zhou et al., 2020; Paules et al., 2020). Currently, research on development of vaccines against SARS-CoV-2 is ongoing worldwide, and interest in effective SARS-CoV-2 therapeutics is increasing rapidly (Wang et al., 2020; Lederer et al., 2020). To succeed in development of a therapeutic or vaccine against SARS-CoV-2, knowledge of the exact target cells where SARS-CoV-2 enter the host and the mechanism of infection in the respiratory tract is essential.

The cellular infectivity of a respiratory virus to the respiratory epithelium depends on the distribution of receptors and activity of host serine proteases in the respiratory tract (Laporte and Naesens, 2017; Bertram et al., 2010; Dittmann et al., 2015). It is becoming increasingly apparent that nasal epithelial cells are the primary target of SARS-CoV-2, and the nasal epithelium is regarded as a portal for initial infection and/or transmission of SARS-CoV-2 to the respiratory tract (Sungnak et al., 2020; Blanco-Melo et al., 2020). SARS-CoV-2 employs angiotensin converting enzyme 2 (ACE2) as a receptor for internalization, and the binding affinity of the spike (S) protein of SARS-CoV-2 to ACE2 was found to be a major determinant of SARS-CoV-2 nasal epithelium cellular infection (Onabajo et al., 2020; Stanifer et al., 2020). Host proteases are involved in cellular invasion by SARS-CoV-2, and transmembrane serine protease 2 (TMPRSS2) is indicated as the principal host protease to mediate cleavage of SARS-CoV-2 S protein in nasal epithelial cells (Sungnak et al., 2020). In this regard, it is of immediate interest to determine whether localized suppression of ACE2 and TMPRSS2 in the nasal epithelium restricts cellular invasion of SARS-CoV-2 and inhibits viral transmission to the respiratory tract.

Inhaled pathogens including respiratory viruses encounter the host immune system through the nasal passage, and the microbial characteristics of the nasal mucus directly impact initiation of the innate immune response (Segal et al., 2014; Kim et al., 2019). Thus, insights into the human nasal mucus microbiome can provide fundamental information regarding defense mechanisms against respiratory virus infection and understanding of microbiome-regulated immune factors contributes to discovery of new concepts of viral infection control (Planet et al., 2016).

Our previous study identified *Staphylococcus epidermidis* as the most abundant constituent in human nasal mucus and showed that *S. epidermidis* induced a potent antiviral defense mechanism in nasal epithelium via interferon-related immune responses (Kim et al., 2019). In this study, we aimed to elucidate another capacity of *S. epidermidis* to potentiate antiviral innate immune responses against SARS-CoV-2 and investigated whether *S. epidermidis* might decrease the expression of SARS-CoV-2 host entry factors in nasal epithelium. Our findings revealed that both *ACE2* and *TMPRSS2* transcriptions were significantly reduced in human nasal epithelium in response to *S. epidermidis*. In addition, the presence of *S. epidermidis* in human nasal mucosa showed negative correlation with gene expression of *ACE2* and *TMPRSS2*. Our findings also showed that *ACE2* transcription was significantly reduced in human nasal epithelium in response to *S. aureus* inoculation. The current findings provide evidence that nasal commensal *Staphylococcus species* create a cellular environment lacking host entry factors of SARS-CoV-2 in human nasal mucosa, which is the main target for invasion of SARS-CoV-2 and these findings can provide a biological antiviral arsenal against invading SARS-CoV-2 through the alteration of host entry factors in favor of host.

## Results

### Nasal epithelium might be a main route of SARS-CoV-2 invasion

**Figure 1:**
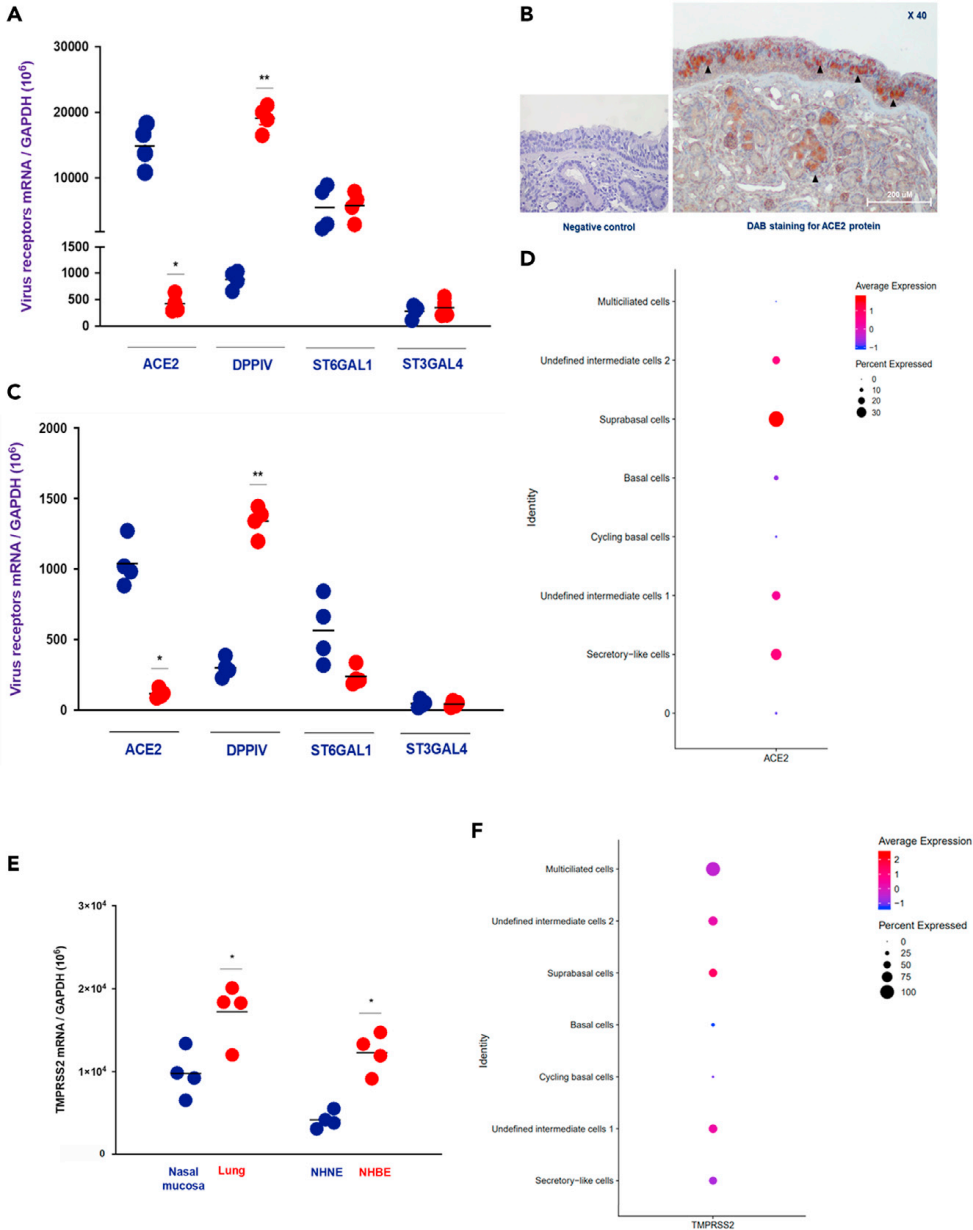
Expression of *ACE2* in human nasal mucosa and lung tissue (A) mRNA levels of *ACE2*, *DPPIV*, *ST6GAL1,* and *ST3GAL4* in human nasal mucosal tissues (blue circles, N = 4) and lung parenchymal tissues (red circles, N = 4) of healthy subjects. (B) Immunohistochemistry (IHC) analysis of ACE2 protein using DAB (3,3′-diaminobenzidine) chromogen was performed in nasal mucosa section from healthy human middle turbinate. Black arrows indicate the positive ACE2 staining in nasal mucosa including epithelium and subepithelial regions (original magnification ×40). (C) mRNA levels of *ACE2*, *DPPIV*, *ST6GAL1,* and *ST3GAL4* in cultured NHNE cells (blue circles, N = 4) and NHBE cells (red circles, N = 4). (D) Expression of *ACE2* from single-cell RNA sequencing (scRNA-seq) of NHNE cells. Dot size represents the proportion of *ACE2* RNA expression within the respective cell type expressing the gene, and dot color represents the average *ACE2* RNA expression level in the particular cell type. (E) mRNA level of *TMPRSS2* in nasal mucosal tissues (blue circles, N = 4), lung parenchymal tissues (red circles, N = 4) of healthy subjects, cultured NHNE cells (blue circles, N = 4) and NHBE cells (red circles, N = 4). (F) Expression of *TMPRSS2* from single-cell RNA sequencing (scRNA-seq) of NHNE cells. The results of real-time PCR are presented as the mean of human tissue or cultured epithelial cells from four subjects, and the IHC imaging is representative of four independent experiments. Gene expression was normalized to GAPDH. ∗p < 0.05, ANOVA with post hoc.

We evaluated *ACE2* RNA expression in human nasal mucosa (N = 4) and lung tissue (N = 4) and compared it to that of *DPPIV*, which encodes a known viral receptor for MERS-CoV, and *ST6GAL1* and *ST3GAL4,* which are important for synthesis of α(2,6)-linked and α(2,3)-linked sialic acids recognized by influenza virus (Jeon et al., 2020; Broszeit et al., 2019). Real-time PCR revealed that mRNA expression of *DPPIV* in lung parenchymal tissue was significantly higher than in the nasal mucosa, and neither mRNA expression of *ST6GAL1* or *ST3GAL4* was significantly different in human nasal mucosa and lung tissue. Unlike the expression of other viral receptors, the mean level of *ACE2* mRNA was higher in the human nasal mucosa (1.7 × 10^9^) than in lung tissue (4.8 × 10^7^) (Figure 1A). Immunohistochemistry (IHC) for ACE2 protein was performed using human nasal mucosa of middle turbinate to determine whether ACE2 protein is mainly present in nasal mucosa (Figure S1). Although the expression of ACE2 protein was observed in a part of submucous gland, IHC results showed that positive DAB (3,3′-diaminobenzidine) staining of ACE2 protein was highly increased in the nasal epithelium relative to the subepithelial area of the human nasal mucosa (Figure 1B).

To further characterize the expression of viral receptors in nasal epithelial cells, we examined *ACE2*, *DPPIV*, *ST6GAL1,* and *ST3GAL4* expression within air-liquid interface cultures of normal human nasal epithelial (NHNE) cells, and the results were compared to those of normal human bronchial epithelial (NHBE) cells. Based on results using this *in vitro* system, we confirmed increased *ACE2* mRNA expression in nasal epithelial cells, and mRNA level of *ACE2* was higher in NHNE than NHBE cells (Figure 1C). We found that *DPPIV* expression was higher in NHBE cells, and no significantly different expression of *ST6GAL1* and *ST3GAL4* was observed between NHNE and NHBE cells. To clarify the cell subsets targeted by SARS-CoV-2 in the human nasal epithelium, we investigated gene expression of *ACE2* depending on nasal epithelial cell subset through single-cell RNA sequencing (scRNA-seq) (Figure S2). We confirmed increased normalized *ACE2* expression in both suprabasal cells and secretory-like NHNE cells (Figure 1D). As a next step, we determined the gene expression level of *TMPRSS2* in human nasal mucosa, lung tissue, NHNE and NHBE cells. Real-time PCR results revealed that the gene expression of *TMPRSS2* was rather lower in human nasal mucosa and NHNE cells than lung and NHBE cells (Figure 1E) and scRNA-seq data showed that *TMPRSS2* gene expression across cell types of NHNE cells was much stronger with a certain specificity for suprabasal cells (Figure 1F). Although principal serine protease expression for SARS-CoV-2 invasion was relatively lower, the cellular receptor required for SARS-CoV-2 to enter the human respiratory tract was more distributed in human nasal mucosa. These data suggest that the nasal epithelium is the primary target of SARS-CoV-2 invasion, and SARS-CoV-2 infection is spread to the respiratory tract after intracellular entry via nasal epithelial cells.

### The expression of SARS-CoV-2 entry-associated genes is altered by human nasal commensal, *S. epidermidis*

**Figure 2:**
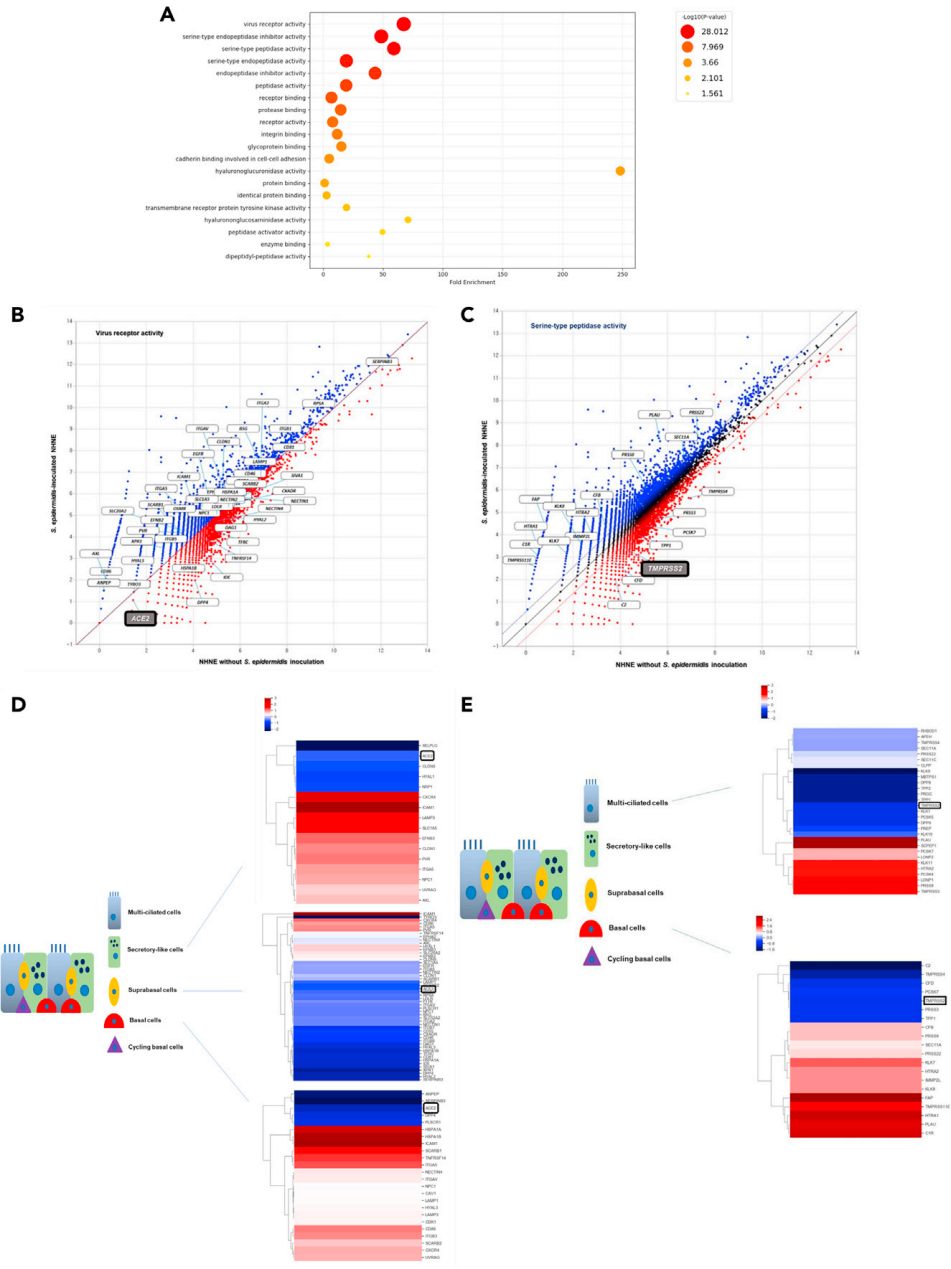
Host transcriptional response to *S. epidermidis* in NHNE cells NHNE cells from four healthy volunteers were inoculated with human nasal *S. epidermidis* at an MOI of 0.25, and total RNA was harvested after 24 h. (A) Dot plot visualization of enriched Gene Ontology (GO) terms related with (A) “serine-type peptidase activity” in *S. epidermidis*-inoculated NHNE cells. (B and C) Scatter plots indicating enriched genes related with (B) virus receptor activity, and (C) serine-type peptidase in *S. epidermidis*-inoculated NHNE cells. (D and E) Heatmap depicting the expression levels of genes related with (D) virus receptor activity and (E) serine-type peptidase activity differentially expressed in *S. epidermidis*-inoculated NHNE cells depending on cellular subset.

We next sought to explore the correlation between the abundant nasal commensal *S. epidermidis* and entry factors of SARS-CoV-2 in the nasal epithelium, NHNE cells from five healthy subjects were inoculated with *S. epidermidis* isolated from healthy human nasal mucus, for 24 h at a multiplicity of infection (MOI) of 0.25 (Figure S3). We performed Gene Ontology (GO) enrichment analysis of scRNA-seq data using cell lysates from the *S. epidermidis*-inoculated NHNE cells to confirm the effect of *S. epidermidis* in restricting host entry factors of SARS-CoV-2. The terms associated with serine-type peptidase activity were also examined, and the results revealed top significant terms of “virus receptor activity,” “serine-type endopeptidase inhibitor activity,” “serine-type peptidase activity,” “peptidase activity,” and “receptor binding” (Figure 2A).

Significant gene populations (fold change ≥ or <1.5 and normalized data (log2) ≥ or <2.0) in *S. epidermidis*-inoculated NHNE cells were compared to those from NHNE cells without *S. epidermidis* inoculation. The scatter plot data of genes associated with virus receptor activity revealed lower *ACE2* gene expression in *S. epidermidis*-inoculated NHNE cells relative to the control (Figure 2B). We also analyzed significant gene expression associated with serine-type peptidase activity and scatter plot data showed that *PLAU*, TMPRSS11E, *KLK7*, *KLK8*, and *HTRA2* mRNA levels were higher in *S. epidermidis*-inoculated NHNE cells. However, the principal host protease for SARS-CoV-2 invasion, *TMPRSS2* transcription was reduced in NHNE cells after *S. epidermidis* inoculation (0.59-fold decrease) relative to the control (Figure 2C).

Next, we analyzed the change of *ACE2* and *TMPRSS2* expression in NHNE cell subsets of basal cells, secretory-like cells, undefined intermediate cells, suprabasal cells, and multiciliated cells in the presence or absence of *S. epidermidis*. Heatmap data depicting genes classified into virus receptor activity revealed that decrease in *ACE2* transcription in response to *S. epidermidis* inoculation was most pronounced in secretory-like, basal, and suprabasal cells and the greatest *ACE2* reduction in response to *S. epidermidis* was observed in basal NHNE cells (Figures 2D and S4).

Based on dot plot data, normalized *TMRPSS2* expression was significantly higher in suprabasal cultured NHNE cells, and the heatmap of serine-type peptidase transcript activity also showed that *TMPRSS2* transcription was significantly reduced in multiciliated and basal NHNE cells in response to *S. epidermidis*-inoculation (Figure 2E). Based on these findings, we suggest that human nasal commensal *S. epidermidis* reduced gene expression associated with host entry of SARS-CoV-2, including *ACE2* and *TMPRSS2,* depending on NHNE cell subset.

**Figure 3:**
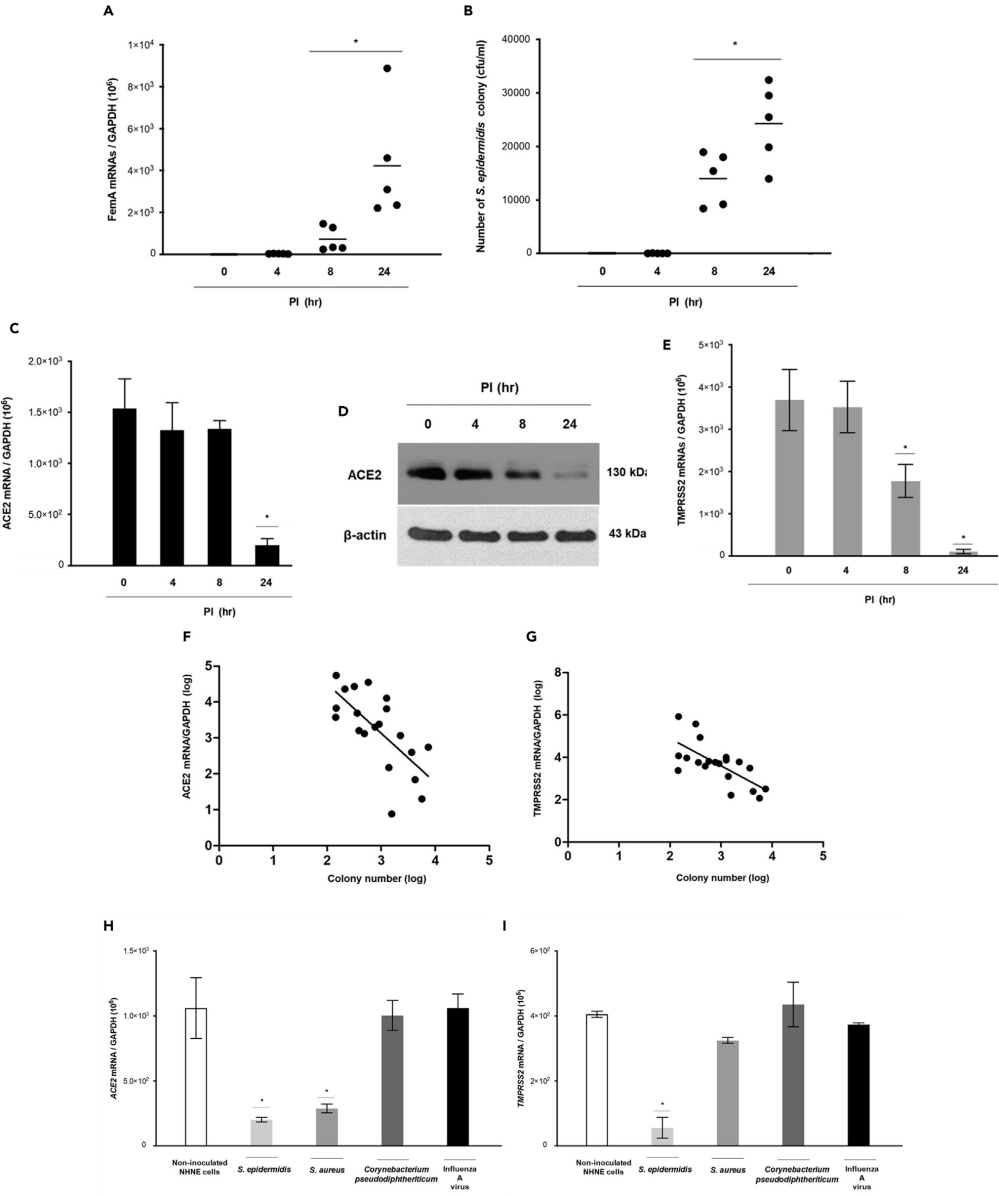
*S. epidermidis* promotes reduction of *ACE2* and *TMPRSS2* expression in the nasal epithelium NHNE cells from five healthy volunteers were inoculated with human nasal *S. epidermidis* at an MOI of 0.25. (A) *femA* mRNA level was monitored by real-time PCR at 0, 4, 8, and 24 h. (B) *S. epidermidis* CFUs were determined over the course of 24 h in the supernatant of *S. epidermidis*-inoculated NHNE cells. (C) *ACE2* mRNA level was determined by real-time PCR until 24 h. (D) *TMPRSS2* mRNA level was determined by real-time PCR at 0, 4, 8, and 24 h in the cell lysate of *S. epidermidis*-inoculated NHNE cells. (E) The intracellular protein levels of ACE2 and TMPRSS2 were measured in the cell lysate of *S. epidermidis*-inoculated NHNE cells until 24 h using western blot analysis. (F and G) The mRNA levels of (F) *ACE2* and (G) *TMPRSS2* in the nasal mucosa from the middle turbinate of healthy volunteers (n = 20), as measured by real-time PCR, were correlated with colony-forming units (CFUs) of *S. epidermidis* isolated from the mucus of the middle turbinate collected from the same subjects. The correlation was determined using Spearman's correlation analysis (ACE2 (Spearman r = −0.7469, *p* = 0.0002) and *TMPRSS2* (Spearman r = −0.6581, *p* = 0.0016). (H and I) *ACE2* and (I) *TMPRSS2* mRNA levels were determined by real-time PCR at 24 h after inoculation of *S. epidermidis*, *S. aureus*, *C. pseudodiphtheriticum*, and influenza A virus (WS/33, H1N1). The western blot is representative of five independent experiments and real-time PCR results are presented as mean ± SD from five independent experiments (Gene expression was normalized to GAPDH. ∗p < 0.05 compared with NHNE cells at 0 hr after inoculation, ANOVA with post hoc).

To better determine the influence of *S. epidermidis* on SARS-CoV-2 host entry factors in the nasal epithelium, NHNE cells from five healthy subjects were inoculated with human nasal *S. epidermidis* at an MOI of 0.25, and then, *S. epidermidis* mRNA level in the cell lysate and the colony count of *S. epidermidis* in the supernatant were assessed until 1-day post infection (dpi). Real-time PCR data revealed that the mean mRNA level of *S. epidermidis femA* increased significantly starting from 8 h post infection (0.8 × 10^9^), and that the highest levels were observed at 1 dpi (4.2×10^9^; Figure 3A). The mean colony forming unit (CFU) of *S. epidermidis* was significantly increased in the supernatant of NHNE cells until 1 day (2.4 × 10^4^ CFU/ml) after *S. epidermidis* inoculation (Figure 3B). Subsequently, we tested whether *S. epidermidis*-inoculated NHNE cells exhibited the decrease of *ACE2* and *TMPRSS2* as shown in scRNA-seq data. Real-time PCR results showed that *ACE2* mRNA was reduced significantly at 1 day after *S. epidermidis* inoculation (Figure 3C) and ACE2 protein expression was also significantly decreased in the cell lysate of *S. epidermidis*-inoculated NHNE cells (Figure 3D). In addition, a gradual decrease of *TMPRSS2* mRNA level was seen in NHNE cells in response to *S. epidermidis*, with the lowest expression observed at 24 h after inoculation (Figure 3E).

Considering the *in vitro* effect of the nasal commensal *S. epidermidis* on entry factors of SARS-CoV-2 in the nasal epithelium, we investigated the relationship between *S. epidermidis* abundance and mRNA levels of *ACE2* and *TMPRSS2* in human nasal mucosa of 20 healthy subjects. Interestingly, *S. epidermidis* CFUs from healthy human nasal mucus was inversely correlated with *ACE2* (Spearman r = −0.7469) and *TMPRSS2* (Spearman r = −0.6581) mRNA levels in human nasal mucosa (Figures 3F and 3G). Through these experimental data, we found that human nasal commensal S. epidermidis transformed nasal epithelial cells into an environment lacking the host entry factors of SARS-CoV-2. These data also indicate that the subjects who have more numbers of *S. epidermidis* in their nasal mucus show relatively lower levels of *ACE2* and *TMPRSS2* gene expression, and that subjects with decreased number of *S. epidermidis* in the nasal mucus have higher transient expression of *ACE2* and *TMPRSS2*.

### Human nasal commensal, *Staphylococcus species* reduces the transcription of SARS-CoV-2 entry-associated genes

We isolated the colonies of each nasal commensal and possessed the 3 species of nasal microbiome including *S. epidermidis (N* = *4)*, *S aureus (N* = *4)*, and *Corynebacterium pseudodiphtheriticum (N* = *3)* (Kim et al., 2019)*.* To examine the effect of nasal commensals on the antiviral immune response, NHNE cells were inoculated with three species of nasal microbiome *S. epidermidis, S. aureus, C. pseudodiphtheriticum* (MOI = 0.25) and influenza A virus (A/WS/33, MOI = 1.0) and both *ACE2* and *TMPRSS2* gene expressions were determined until 24 h after inoculation. Intriguingly, the current data showed *S. epidermidis* and *S. aureus* inoculation reduced the transcriptions of *ACE2* in nasal epithelium but *C. pseudodiphtheriticum* and influenza A virus did not exhibit any significant reduction of *ACE2* mRNA level in nasal epithelial cells (Figure 3H). Real-time PCR results revealed that *TMPRSS2* mRNA level was significantly reduced in NHNE cells in response to *S. epidermidis* inoculation. *S. aureus* also reduced *TMRPSS2* mRNA level at 24 hr after inoculation but it did not seem to significantly reduce compared to the decrease of *ACE2* gene expression in NHNE cells (Figure 3I). Collectively, these findings showed that human nasal microbiome *S. aureus* also decrease the transcription of SARS-CoV-2 cellular receptor in nasal epithelium and *Staphylococcus species* nasal commensals might suppress the cellular entry of SARS-CoV-2 at the level of human nasal epithelium.

## Discussion

Our study revealed that *S. epidermidis*, which is abundant in healthy human nasal mucus, might be significantly associated with the reduction of SARS-CoV-2 host entry factors and *S. aureus* also lead to downregulated expression of SARS-CoV-2 cellular receptor in human nasal epithelium. Following inoculation of *S. epidermidis* and *S. aureus*, both ACE2 and TMPRSS2 expressions were reduced in nasal epithelium, thereby skewing the balance of host entry factors in the nasal mucosa in favor of the host. This study highlights the contribution of the nasal commensal *S. epidermidis* to the defense mechanisms against SARS-CoV-2 infection, which mainly targets nasal epithelial cells for cellular invasion.

Human respiratory viruses first encounter host defense mechanisms in the nasal epithelium, and host protection against viral infections can be conferred by a specialized innate immune system of the nasal epithelium capable of combating invasion by respiratory viruses. Recently, growing evidence shows that the entry factors for SARS-CoV-2, including ACE2 and TMPRSS2, are dominantly found in the nasal epithelium, and nasal epithelial cells have been determined as a potential cellular target of SARS-CoV-2 infection (Gallo et al., 2021; Klingenstein et al., 2020). Our data also showed that human nasal mucosa and cultured nasal epithelial cells exhibited higher expression of ACE2 expression, which was more dominant in suprabasal and secretory-like cells in human nasal epithelium. The expression of *TMPRSS2* in nasal epithelial cells was rather lower than in bronchial epithelial cells, but the localization of *TMPRSS2* was relatively stronger in suprabasal cells across cell types of nasal epithelium. Thus, there is an urgent need for investigation of antiviral immune responses restricting the virus entry and its replication in nasal epithelium, ultimately leading to treatment strategies of SARS-CoV-2 infection and we characterized the contribution of the antiviral defense mechanisms in nasal mucosa or nasal epithelium against SARS-CoV-2 invasion.

Nasal symbiotic microorganisms are present in the nasal mucus and are previously shown to play a special role in inducing an antiviral immune response, especially activated in the nasal epithelium. Host protection against viral infections can be conferred by the nasal microbiome via a specialized immune mechanism and recent work has highlighted *S. epidermidis* is capable of combating invasion by respiratory viruses (Kim et al., 2017, 2019; Gallo et al., 2021; Won et al., 2019; An et al., 2018). As pointed out recently, *S. epidermidis* is the most abundant commensal organism in the healthy human nasal mucus, and the presence of *S. epidermidis* strengthens the frontline antiviral immune defense response in the respiratory tract through the modulation of interferon-dependent innate immune mechanisms in the nasal mucosa (Kim et al., 2019). The current findings indicate that the nasal commensal *S. epidermidis* downregulated cellular entry factors in the nasal epithelial cells which support entry of SARS-CoV-2 leading to spread to the respiratory tract. *ACE2* transcription in the nasal epithelium was significantly reduced after inoculation with *S. epidermidis* in suprabasal and secretory-like NHNE cells. In addition, *TMPRSS2* expression was reduced in *S. epidermidis*-inoculated NHNE cells, especially basal and multiciliated cells. This result is underscored by the inverse correlation between *ACE2* and *TMPRSS2* of the nasal mucosa and *S. epidermidis* colony number in human nasal mucus. Thus, a greater abundance of *S. epidermidis* in the nasal mucus results in lower *ACE2* and *TMPRSS2* expression in the nasal mucosa of healthy subjects. We also sought to find how other nasal commensals affect intracellular invasion of SARS-CoV-2 and *S. aureus* reduces ACE2 transcription in nasal epithelium in contrast to *C. pseudodiphtheriticum* and influenza A virus. Therefore, we understand that the reduction of SARS-CoV-2 host entry factors by nasal commensals is mainly caused by *staphylococcus species* in nasal epithelium.

Serine protease inhibitors may be considered an emerging broad therapeutic group against multiple respiratory viruses that rely on these enzymes for their replication, and the broad serine protease inhibitors are known to suppress influenza viral replication through the inhibition of serine proteases (Meyer et al., 2013; Zhou et al., 2020). We presume that targeting suppression of cellular receptors and serine-type proteases in the nasal epithelium shows promise to inhibit SARS-CoV-2 spread from the level of nasal mucosa and intranasal inoculation of *S. epidermidis* may influence SARS-CoV-2 invasion into the nasal epithelium.

Here, we described that the nasal commensal *Staphylococcus species*-regulated transcription of *ACE2* and *TMPRSS2* shapes a cellular environment lacking host entry factors of SARS-CoV-2 in nasal epithelium and downregulation of host entry factors by *S. epidermidis* and *S. aureus* can restrict cellular invasion of SARS-CoV-2 into nasal epithelial cells. Our work highlights the importance of host-bacterial commensalism in shaping the cellular environment of the nasal epithelium resulting in disturbing SARS-CoV-2 invasion into epithelial cells through modulation of host entry factors.

### Limitations of the study

The present study is somewhat limited. We did not perform any experiments with SARS-CoV-2 and did not prove whether *S. epidermidis* and *S. aureus* could reduce the host entry of SARS-CoV-2 to nasal epithelium or not. Furthermore, we did not directly demonstrate the key signaling between reduction of ACE2 and TMPRSS2 expression through *S. epidermidis* and *S. aureus* inoculation in the nasal epithelium. However, we determined that targeting host entry factors might have a strong impact in disturbing the cellular invasion of SARS-CoV-2, thus identifying *S. epidermidis*-reduced ACE2 and TMPRSS2 as an influence on the development of antiviral strategies to suppress SARS-CoV-2 infections. Our study just provides understanding that nasal microbiome *Staphylococcus species* could potentially benefit the host respiratory tract through the downregulation of SARS-CoV-2 host entry factors at the nasal epithelium.

## STAR★Methods

### Key resources table

**Table:** 

REAGENT or RESOURCE	SOURCE	IDENTIFIER
**Antibody**		

ACE2	Cell Signaling Technology	4355

**Biological samples**		

Human nasal epithelial cells	Seoul national university hospital	does not apply
Human bronchial epithelial cells	Seoul national university hospital	
Human nasal mucosa	Seoul national university hospital	
Human lung tissue	Seoul national university hospital	

**Critical commercial assays**		

BCA protein assay kit	Thermo Fisher Scientific	23250

**Deposited data**		

Single-cell RNA sequencing data	GEO	GSE167509

### Resource availability

#### Lead contact

Further information and requests for resources and reagents should be directed to and will be fulfilled by the lead contact, Dr. Hyun Jik Kim (hyunjerry@snu.ac.kr).

#### Material availability

The study did not generate any materials.

### Experimental mode and subject details

#### Ethics statement

Participation was voluntary, with written informed consent obtained from all subjects. This study was approved by the Institutional Review Board (IRB) of the Seoul National University College of Medicine (no. 1709-049-883).

#### Subjects and sample collection

The 1×1-cm-sized nasal mucosal tissue samples from the middle turbinate of the subjects (N = 4) who underwent septoplasty under general anesthesia in the Department of Otorhinolaryngology Seoul National University Hospital (Seoul, Korea) were obtained for real-time PCR. Also, 1×1-cm-sized human lung parenchymal tissues of the subjects (N = 4) were obtained from the subjects referred to the Department of Thoracic and Cardiovascular Surgery Seoul National University Hospital, primarily for pneumonectomy.

#### Nasal mucus nasal commensals characterization

Mucus from the middle turbinate of healthy volunteers was collected individually using sterile 3M Quick swabs (3M Microbiology Products, St. Paul, MN, USA) from four subjects using a rigid 0-degree endoscope in an operating room. The swabs with mucus were fixed in a fixative solution and transported immediately to the laboratory for identification and subsequent microbial analysis. For bacterial colony isolation, the mucus was plated on Lysogeny Broth (LB) plates. After two days of incubation, bacterial colonies were obtained from the LB plates, and the species of each colony was identified using GS-FLX 454 pyrosequencing and 16S rRNA gene amplification (Kim et al., 2019). Four *S. epidermidis,* four *S. aureus,* and three *C. pseudodiphtheriticum* strains were isolated from four individuals.

#### Cell culture

Normal human nasal epithelial (NHNE) cells were cultured as described previously Park et al. (2016). Briefly, passage-2 NHNE cells (1 × 10^5^ cells/culture) were seeded in 0.25 ml of culture medium on Transwell-Clear culture inserts (24.5 mm, with a 0.45-mm pore size; Costar Co., Cambridge, MA, USA). Cells were cultured in a 1:1 mixture of basal epithelial growth medium and DMEM containing previously described supplements. Cultures were grown while submerged for the first 9 days. The culture medium was changed on Day 1 and every other day thereafter. An air–liquid interface (ALI) was created on Day 9 by removing the apical medium and feeding the cultures from the basal compartment only. The culture medium was changed daily after establishment of the ALI. The antifungal agent fungizone (1 ml/1000 ml media) (Life Technologies, Grand Island, NY, USA) was added after filtering the media. All experiments described here used cultured nasal epithelial cells at 14 days after ALI.

#### Single-cell RNA sequencing (scRNA-seq)

Library construction was performed using 10X Chromium Single Cell 3′ reagent kits v3.1. Samples were sequenced using the Illumina NovaSeq 6000 platform, and preliminary sequencing results were converted to FASTQ files using the Cell Ranger pipeline. We followed the 10x Genomics standard sequence protocol by trimming the barcode and unique molecular identifier (UMI) end to 26 bp and the mRNA end to 98 bp. Then, the FASTQ files were aligned to the human reference genome (GRCh38). Subsequently, we applied Cell Ranger for preliminary data analysis and generated a file that contained a barcode table, a gene table, and a gene expression matrix. We used the WinSeurat v2.1 (Ebiogen Inc., Seoul, Korea) based on Seurat version 3 for QC, analysis, and exploration of single-cell RNA-seq data (Butler et al., 2018; Stuart et al., 2019). Data mining and graphic visualization were performed using ExDEGA (Ebiogen Inc., Seoul, Korea).

#### Real-time PCR

NHNE cells were infected with *S. epidermidis* for 4, 8, or 24 h, and total RNA was isolated using TRIzol (Life Technology, Seoul, Korea). cDNA was synthesized from 3 μg of RNA with random hexamer primers and Moloney murine leukemia virus reverse transcriptase (Perkin Elmer Life Sciences, Waltham, MA, USA and Roche Applied Science, Indianapolis, IN, USA). Amplification was performed using the TaqMan Universal PCR master mix (PE Biosystems, Foster City, CA, USA) according to the manufacturer's protocol. Briefly, 12 μl amplification reactions contained 2 μl of cDNA (reverse transcription mixture), oligonucleotide primers (final concentration of 800 nM), and TaqMan hybridization probe (200 nM). Real-time PCR probes were labeled at the 5′ end with carboxyfluorescein (FAM) and at the 3′ end with the quencher 5-carboxytetramethylrhodamine (5-TAMRA). To quantify cellular viral level and host gene expression, cellular RNA was used to generate cDNA. Primers for *femA*, *TMPRSS2*, and *ACE2* were purchased from Applied Biosystems (Foster City, CA, USA). Real-time PCR was performed using the PE Biosystems ABI PRISM® 7700 Sequence Detection System. Thermocycler parameters were as follows: 50°C for 2 min, 95°C for 10 min, and 40 cycles of 95°C for 15 s and 60°C for 1 min. Target mRNA levels were quantified using target-specific primer and probe sets for *femA*, *TMPRSS2*, and *ACE2*. All PCR assays were quantitative and utilized plasmids containing the target gene sequences as standards. All reactions were performed in triplicate, and all real-time PCR data were normalized to the level of glyceraldehyde phosphate dehydrogenase (*GAPDH*, 1×10^6^ copies) to correct for variations between samples.

#### Western blot analysis

Protein level of ACE2 was assessed using western blot analysis, and the monoclonal antibody of ACE2 was purchased from Cell Signaling Technology (Beverly, MA, USA). The NHNE cells and were lysed with 2X lysis buffer (250 mM Tris-Cl (pH6.5), 2% SDS, 4% β-mercaptoethanol, 0.02% bromophenol blue, and 10% glycerol). Cell lysate (30 μg of protein) was electrophoresed in 10% SDS gels and transferred to polyvinylidene difluoride membranes in Tris-buffered saline (TBS; 50 mM Tris-Cl (pH 7.5), 150 mM NaCl) for 1 h at room temperature. The membrane was incubated overnight with primary antibody (1:500) in Tween-Tris buffered saline (TTBS; 0.5% Tween-20 in TBS). After washing with TTBS, the blot was incubated for 1 h at room temperature with secondary anti-rabbit antibody (1:1,000, Cell Signaling, Beverly, MA, USA) in TTBS and was visualized using an ECL system (Amersham, Little Chalfont, UK).

#### Immunohistochemistry

Human nasal mucosa was obtained from the middle turbinate of healthy subjects who had nasal surgery due to nasal obstruction, and paraffin block slides were prepared for immunostaining. Immunohistochemical analysis was performed using ACE2 antibody (1:200, Cell Signaling Technology, MA, USA) to determine protein level in the nasal mucosa. Briefly, 5-μm sections were fixed in acetone for 10 min at room temperature (RT). Non-specific protein staining was blocked with goat serum. Slides were treated with 0.5% hydrogen peroxidase to eliminate endogenous peroxidase for 10 min at RT and incubated with primary antibody overnight at RT. After washing with Tris-buffered saline (TBS, pH 7.5), slides were incubated with horseradish peroxidase-conjugated secondary antibody (Thermo, Asheville, NC, USA) for 30 min at RT. Chromogen (3-amino-9-ethylcarbazole) was applied for visualization. Glass cover slides were mounted and examined with optical microscopy and ACE2 protein was detected with DAB (3,3′-diaminobenzidine) chromogen staining. The same procedures were performed using non-immunized mouse IgG (purified IgG, Sigma) instead of primary antibody as a negative control.

### Quantification and statistical analysis

Gene expression from human nasal mucosa, lung tissue, NHNE and NHBE cells are presented as mean value. For *in vitro* study, at least three independent experiments were performed with cultured cells from each donor, and the results are presented as the mean value ±standard deviation (SD) of triplicate cultures. Differences between groups were evaluated by analysis of variance (ANOVA) with a *post hoc* test. Associations between *ACE2*, *TMPRSS2*, and *S. epidermidis* colonies in human nasal mucosa were evaluated using Spearman's correlation analysis. Statistical analyses were performed using GraphPad Prism software (version 8.0; GraphPad Software, La Jolla, CA, USA). Differences were considered significant at *p* value < 0.05.

## Data Availability

The original scRNA-seq data of the NHNE cells with *S. epidermidis* inoculation are available at https://www.ncbi.nlm.nih.gov/geo/query/acc.cgi?acc=GSE167509.
