# Supplementary material for: The nasal symbiont Staphylococcus species restricts the transcription of SARS-CoV-2 entry factors in human nasal epithelium
Source: iScience. 2021 Sep 25;24(10):103172. doi: 10.1016/j.isci.2021.103172 (PMC8464034; doi:10.1016/j.isci.2021.103172)
Supplement: Document S1. Figures S1–S4 [file mmc1.pdf]

iScience, Volume *24*

Supplemental information

The nasal symbiont *Staphylococcus species* restricts the transcription of SARS-CoV-2 entry factors in human nasal epithelium

Jeong-Yeon Ji, Ara Jo, Jina Won, Chan Hee Gil, Haeun Shin, Sujin Kim, Yung Jin Jeon, and Hyun Jik Kim

Supplemental information

The nasal symbiont *Staphylococcus species* restricts the transcription of SARS-CoV-2 entry factors in human nasal epithelium

Jeong Yeon Ji, Ara Jo, Jina Won, Chan Hee Gil, Haeun Shin, Sujin Kim, Yung Jin Jeon, Hyun Jik Kim

Healthy human nasal mucosa

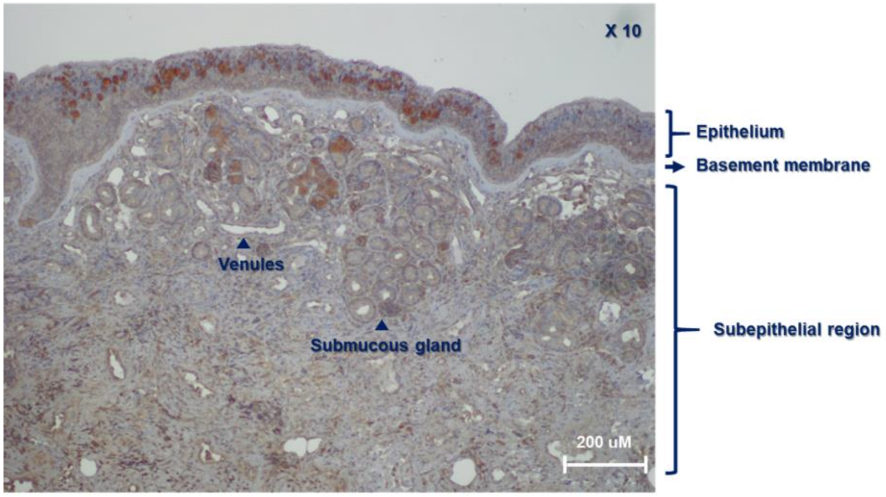

DAB staining for ACE2

Figure S1. Immunohistochemistry (IHC) for ACE2 protein in nasal mucosa. Related Figure 1B

IHC analysis of ACE2 protein using DAB chromogen was performed in nasal mucosa section from healthy human middle turbinate. Black arrows indicate the positive ACE2 staining in nasal mucosa including epithelium and subepithelial regions (original magnification x10).

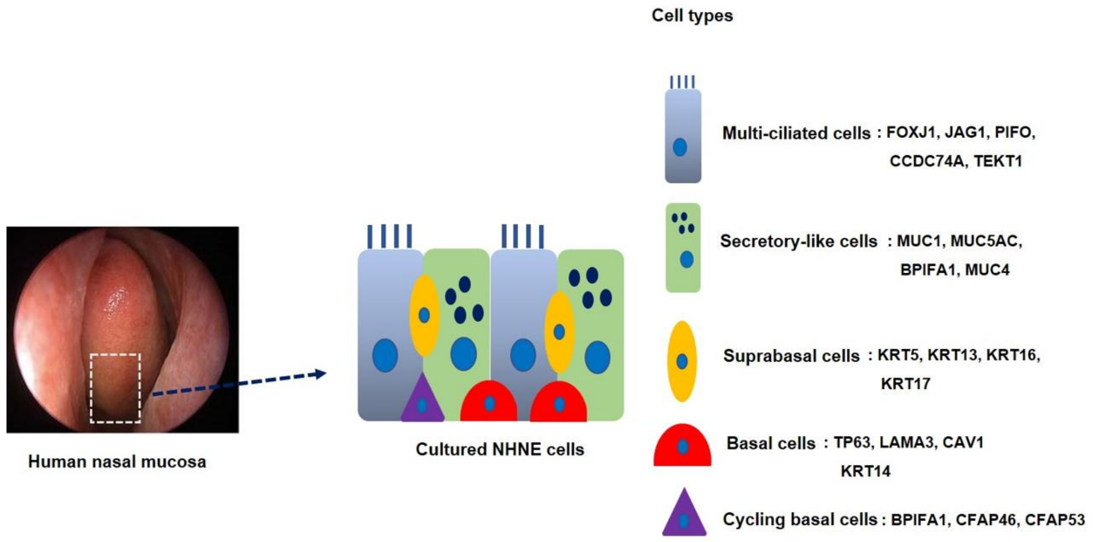

Figure S2. Schematic illustration depicts the sampling location of surgical nasal mucosa specimen (middle turbinate) for primary nasal epithelial culture and overview of the major cell types in the nasal epithelial cells for single cell RNA sequencing. Related Figure 1D

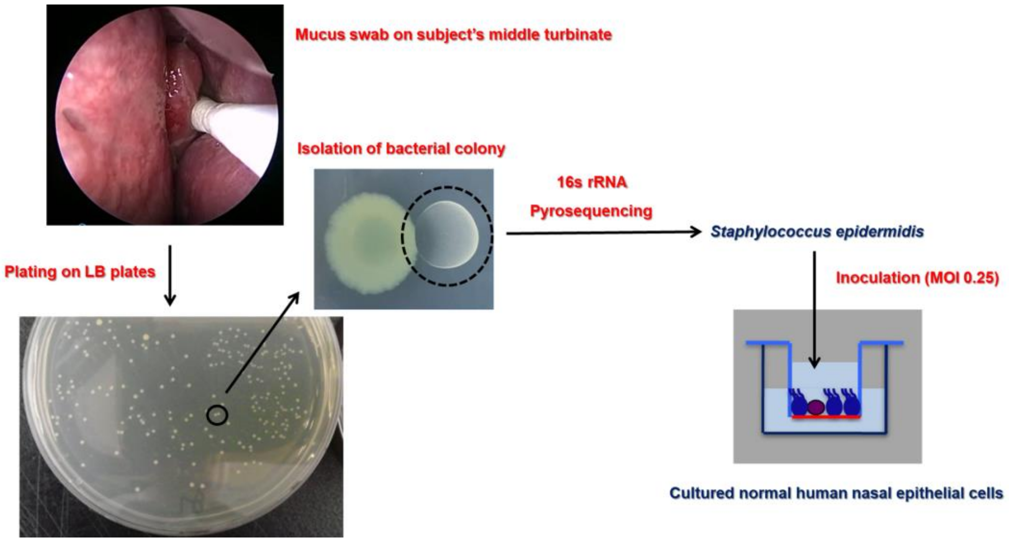

Figure S3. Schematic illustration of step-by-step experiments from *S. epidermidis* isolation to inoculation of NHNE cells. Related Figure 2A

For bacterial colony isolation, nasal mucus of healthy subjects was obtained using a cotton swab and was used to inoculate Lysogeny Broth (LB) plates. After 2 days of incubation, bacterial colonies were obtained from the LB plates, *S. epidermidis* colonies were identified using GS-FLX 454 pyrosequencing by 16S rRNA gene amplification, and *S. epidermidis* was used to inoculate NHNE cells (MOI 0.25).

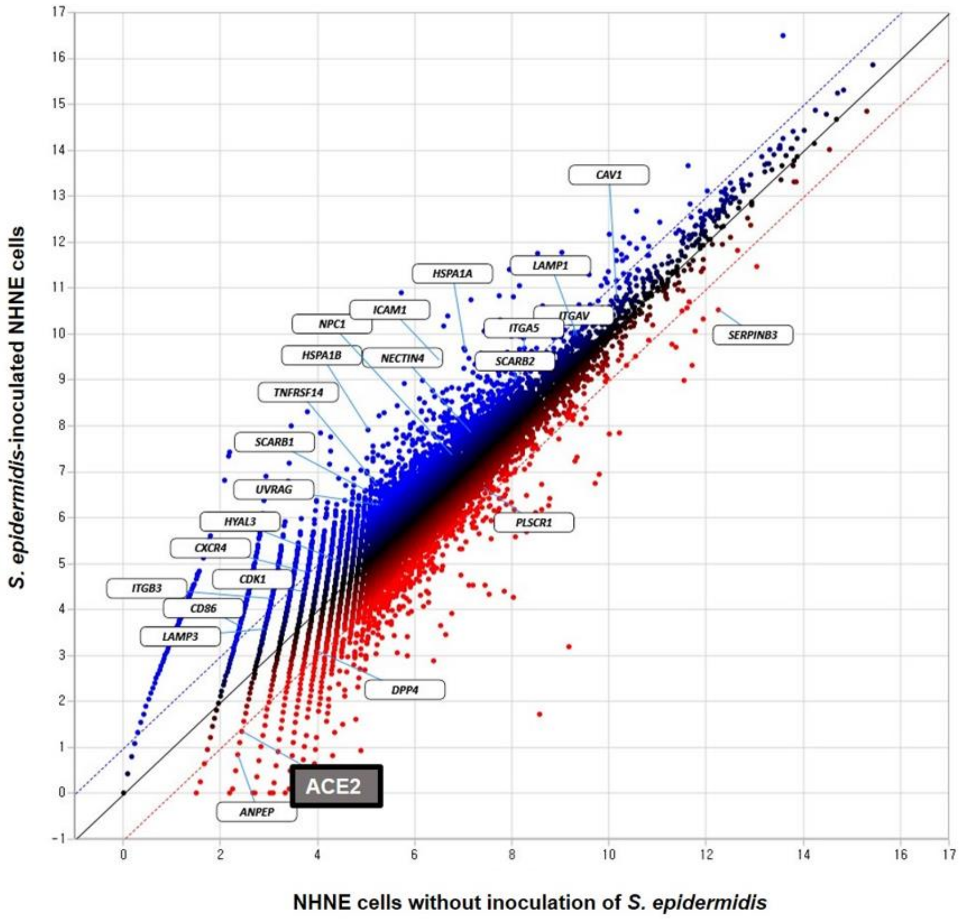

Figure S4. Host transcriptional response to *S. epidermidis* in NHNE cells. Related Figure 2D

Scatter plots indicating enriched genes related with virus receptor activity, in *S. epidermidis*-inoculated basal NHNE cells.
